# Inhibition of *BRUTUS* Enhances Plant Tolerance to Zn Toxicity by Upregulating Pathways Related to Iron Nutrition

**DOI:** 10.3390/life12020216

**Published:** 2022-01-30

**Authors:** Yaxin Zhu, Yujie Dai, Xiangting Jing, Xingxing Liu, Chongwei Jin

**Affiliations:** College of Natural Resources and Environmental Science, Zhejiang University, Hangzhou 310058, China; 21714110@zju.edu.cn (Y.Z.); 21714139@zju.edu.cn (Y.D.); 22114124@zju.edu.cn (X.J.)

**Keywords:** Zn phytoremediation, iron nutrition, metal ions homeostasis, biotechnological method

## Abstract

The identification of the key genes regulating plant tolerance to Zn stress is important for enhancing the Zn phytoremediation of targeted plants. Here, we showed that the T-DNA insertion-induced inhibition of the *BRUTUS* (*BTS*) gene in the *bts-1* mutant greatly improved Zn tolerance, as indicated by increased biomass production and reduced leaf chlorosis. The *ProBTS::BTS-GFP* complementation in the *bts-1* mutant abolished the improvement of Zn tolerance. Unexpectedly, the *bts-1* mutant had higher and comparable Zn concentrations in the roots and citrate effluxer shoots, respectively, compared to wild-type plants. As a result, the shoots and roots of *bts-1* mutants had 53% and 193% more Zn accumulation than the wild-type plants, respectively. RNA-seq analyses revealed that the Fe nutrition-related genes were upregulated in *bts-1* mutants, especially under Zn stress conditions. Therefore, the *bts-1* mutants had a greater Fe concentration and a higher Fe/Zn ratio than the wild-type plants exposed to Zn toxicity. Further study showed that the differences in Zn tolerance between *bts-1* and wild-type plants were minimized by eliminating Fe or supplementing excessive Fe in the growth medium. Taken together, the T-DNA insertion-induced inhibition of BTS improves plant Zn tolerance by optimizing Fe nutrition; thus, the knockdown of BTS may be a promising approach for improving Zn phytoremediation efficiency.

## 1. Introduction

Generally, the average soil zinc (Zn) concentration varies from 10 to 300 mg/kg [1]. However, owing to rapid industrialization, mining, sewage irrigation, and the application of agrochemicals, the pollution of soils with Zn has greatly increased over the past decades [1]. The Zn concentration can reach more than 1000 mg/kg in polluted soils around industrial areas, which poses a severe threat to human health and the ecosystem. Therefore, it is imperative to develop efficient and environmentally friendly measures to restore Zn-contaminated soils. Currently, phytoremediation, the use of plants to remedy contaminated soils, is regarded as an important strategy because of its convenient, cost-effective, and environment friendly features [2,3]. Several Zn-hyperaccumulated plants have been identified [4,5,6], but most of these plants have slow growth and low biomass, which limits their efficacy in remediating contaminated soils [7,8,9]. As an alternative, biotechnological pathways could be adopted to genetically improve the Zn tolerance of the plants with fast growth and large biomass [10,11,12]. Therefore, the identification of key genes regulating Zn tolerance in plants is an important theoretical aspect for improving the phytoremediation efficiency of targeted plants, which also requires a more complete understanding of the molecular mechanisms underlying Zn uptake and tolerance in plants.

Although Zn is an essential micronutrient required for plant growth and development [13,14], excess Zn can also be harmful because it may affect the function of other metal ions in plants. For example, Zn can replace other divalent cations, such as Fe, Mg, and Mn, which are involved in the proper functioning of a number of photosynthetic enzymes. As a result, Zn over-accumulation in plants often results in lower photosynthetic rates, photo-oxidative damage, and visible impeded plant growth [14,15,16]. Therefore, the interactions between Zn and other divalent cations have been of great interest [17,18,19,20]. This is particularly true for the relationship between Zn and Fe. Fe deficiency promotes the absorption of Zn by upregulating the expression of Fe nutrition-related genes, such as *IRT1/IRT3* and *YS1/YSL*, as well as several chelators including mugneic acids (MAs), phytic acid (PA), citrate, and nicotianamine (NA) [21,22,23,24,25,26]. On the other hand, excess zinc supplementation in the growth medium would also result in Fe deficiency in the plants, probably due to competition between Zn and Fe during their uptake by root cells and their translocation to plant tissues [23,27]. Considering the above interaction between Zn and Fe, manipulating the genes that regulate plant Fe nutrition by using biotechnological pathways might be a strategy to improve Zn tolerance in plants.

Among the multiplex Fe regulators in plants, the function of BTS is gradually concerned due to its sophisticated capacity for Fe regulation [28,29,30]. BTS consists of several conserved domains, including three hemerythrin (HHE) cation-binding domains located near the N terminus, a CHY zinc-finger domain, and a Really Interesting New Gene (RING) domain near the C terminus [29]. BTS was proposed as a potential Fe sensor because its HHE domain could bind to iron ions [28,31]. The RING domain, which has an E3 ligase capability, was shown to be involved in the 26S proteasome-mediated ubiquitination degradation of several factors involved in regulating iron uptake and homeostasis [28,32]. Consequently, the knockdown of BTS was shown to significantly improve Fe nutrition in plants [28,29]. Considering the interaction of Fe and Zn in plants, the potential efficacy of partial-loss-of BTS function in improving Zn phytoremediation was evaluated in this study by using T-DNA inserted in an *Arabidopsis thaliana* mutant [28]. We demonstrated that in Zn-contaminated medium, *BTS* inhibition not only enhanced Zn tolerance of plants, but also increased Zn accumulation by improving the Fe nutrition of plants.

## 2. Materials and Methods

### 2.1. Plant Materials

The *Arabidopsis* mutants *bts-1* (SALK_016526), *bts-2*, *fro2*, and *frd3* (CS8506) were in the Col-0 background. The *bts-1* and *frd3* mutants were obtained from the Arabidopsis Biological Resource Center (ABRC). The *bts-2* and *fro2* mutants were kindly gifted by Hongbin Wang (Sun Yat-sen University, Guangzhou, China) and Wolfgang Busch (Salk Institute for Biological Studies, La Jolla, CA, USA) [30,33,34], respectively. The *Pro BTS::BTS-GFP* (*BBG*) transgenic plant line was generated by ligating the *BTS* native promoter and coding sequence into the pCAMBIA1300 vector and transforming the constructed plasmid into *bts-1* plants with *Agrobacterium*-mediated floral dip method [35]. Primers used for genotyping and cloning are listed in Table 1.

### 2.2. Plant Cultivation

The seeds were sterilized using 25% NaClO, washed three times with sterilized water, and then germinated on the agar medium containing the nutrients as previously described [36]. Conditions in the growth room were as follows: a 12 h/12 h light-dark cycle, 80% humidity, and light intensity of 150–200 µmol photons m^−2^s^−2^. After 3 days of germination, the seedlings were transferred to agar medium with normal Zn supply (0.5 µM ZnSO_4_) or different doses of Zn (100, 200, 300, 400, 500 µM ZnCl_2_). For the series of Fe treatments, 0, 10, 50, and 200 µM Fe-EDTA was added. The other nutrient ingredients and pH were the same as those of the basal agar medium. Because the Fe uptake and transport in *fro2* and *frd3* mutants were severely impaired, the Fe concentration needed to be raised to 100 µM to maintain the normal growth of *fro2* and *frd3* mutants.

### 2.3. Chlorophyll Quantification

The leaves of *Arabidopsis thaliana* (0.1 g) were sampled from the treatments with the normal Zn (0.5 µM ZnSO_4_) or the 300 μM ZnCl_2_ stress. The chlorophyll in leaves was extracted using 3 mL of 80% acetone, and the absorbance of the acetone solution at 645 nm and 663 nm was recorded with a spectrophotometer (SP-1920, Shanghai, China). The chlorophyll concentration was calculated as previously described [37].

### 2.4. Measurement of Zn and Fe Contents

The root samples were desorbed for 30 min with 0.5 mM CaCl_2_ and then rinsed three times with deionized water. The shoot samples were thoroughly rinsed three times with deionized water. The tissue samples were then dried for 72 h at 65 °C, weighed, and then digested with HNO_3_ as described by Jin et al. [38]; subsequently, the Fe and Zn contents were analyzed using a 4200 MP-AES (Agilent Technologies, USA).

### 2.5. RNA Isolation and Transcription Analysis

Total RNA of root tissues was isolated using RNAiso Plus (TaKaRa, Otsu, Japan), and complementary cDNA was synthesized using the PrimeScript RT reagent kit (TaKaRa, Otsu, Japan). qRT-PCR analyses were performed using a SYBR Green RT-PCR kit (TaKaRa, Otsu, Japan) and measured using an ABI StepOnePlus (ABI, Los Angeles, CA, USA). The primers are listed in Table 1, and relative transcript abundance was calculated using the comparative cycle threshold, normalized to *AtUBQ10* [36].

### 2.6. RNA Sequencing and Functional Enrichment Analysis

Total RNA was extracted from the roots of Col-0 and *bts-1* plants using MagZol Reagent (Magen, Shanghai, China) according to the manufacturer’s protocol. The NEBNext Ultra RNA Library Prep Kit for Illumina (New England Biolabs, MA, USA) was used for library preparation. Sequencing was carried out with a HiSeq Xten sequencer (Illumina) at RIBOBIO (Guangzhou, China). Raw sequencing reads were quality controlled and trimmed using Trimmomatic tools and FastQC. Clean read mapping to the *A. thaliana* TAIR10 reference genome and transcript assembly were performed using HISAT2 and StringTie [39]. Significantly differentially expressed genes were assessed according to an adjusted *p*-value threshold of <0.05 and |log_2_(fold change)| of >1 using Ballgown [39]. GO enrichment analyses of DEGs were performed using the R package ClusterProfile [40]. Heatmaps were generated using the R package ComplexHeatmap [41].

### 2.7. Statistical Analysis

The statistical significance of all data was determined using analysis of variance (ANOVA), followed by Duncan’s multiple range test. Statistical significance was set at *p* < 0.05.

## 3. Results

### 3.1. Effects of BTS Inhibition on Zn Tolerance and Zn Accumulation

**Figure 1:**
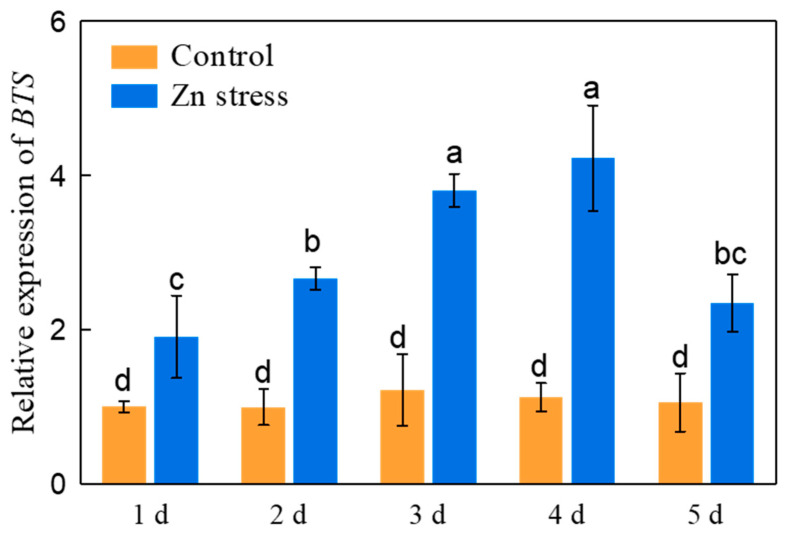
Effect of Zn stress on the expression of *BTS* in roots of wild-type Col-0. The seeds were germinated on basal agar medium as described in the Materials and Methods section. 3-day-old seedlings were transferred to normal Zn or 300 µM Zn-contained agar media. The expression of *BTS* in roots was analyzed after 1,2,3,4,5 days of transfer. *AtUBQ10* was used as a housekeeping gene. Bars represent the standard deviation (*n* = 5). Different letters above the bars indicate significant differences (*p* < 0.05).

The homozygosity of the T-DNA insertion mutant *bts-1* was verified by genomic PCR analysis (Figure S1 in Supplementary Materials). We first compared the growth of the *bts-1* mutant and WT in various Zn treatments (100–500 μM). As shown in Figure S2, the *bts-1* mutant exhibited similar growth under normal growth conditions to the WT, but showed better growth in the medium containing 200–400 μM Zn (Figure S2). Furthermore, we found that the *ProBTS::BTS-GFP* complementation in the *bts-1* mutant abolished the improvement of Zn tolerance (Figure S2). Another *BTS* mutant, *bts-2*, which has a 47 bp fragment deletion in the first intron also showed the enhanced Zn tolerance (Figure S2). However, the *bts-2* mutant showed poor growth under normal condition. Therefore, we used the *bts-1* mutant to study the role of BTS in Zn tolerance in the remaining studies. These results indicate that the inhibition of *BTS* is indeed beneficial for improving Zn tolerance. Given that the growth difference between the WT and the *bts-1* mutant was more obvious in 300 μM Zn than in the other Zn doses (Figure S2), the 300 μM Zn treatment was used in the remaining studies. In this condition of Zn stress, the expression of *BTS* was significantly upregulated. The upregulation was gradually increased along with the increase of Zn exposure time, and the greatest upregulation was found on the fourth day of Zn stress (Figure 1). The results suggested that *BTS* may negatively regulate the tolerance to Zn stress in plants.

**Figure 2:**
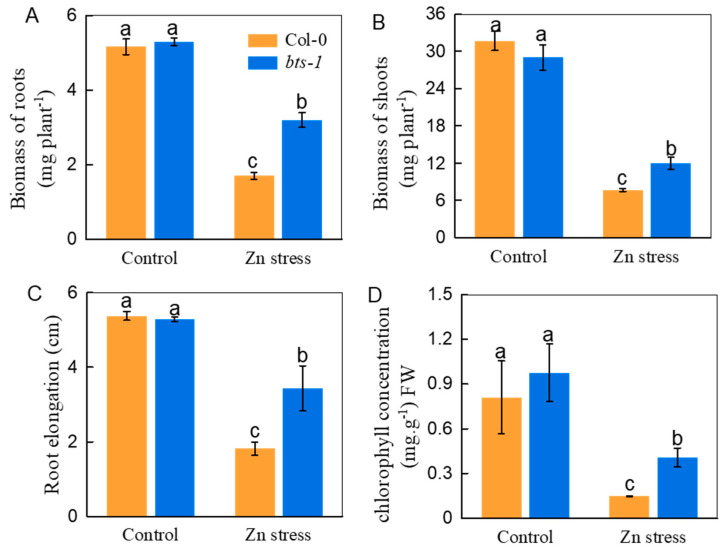
Tolerance of wild-type Col-0 and *bts-1* mutants to Zn stress. After germination on basal agar medium and growth for 3 days, all lines were transferred to normal Zn or 300 µM Zn-contained plates for growing for another 5 days. (**A**,**B**) Biomass of roots and shoots, respectively, (**C**) root elongation, and (**D**) chlorophyll concentrations were analyzed. Bars represent the standard deviation (*n* = 5). Different letters above the bars indicate significant differences (*p* < 0.05).

**Figure 3:**
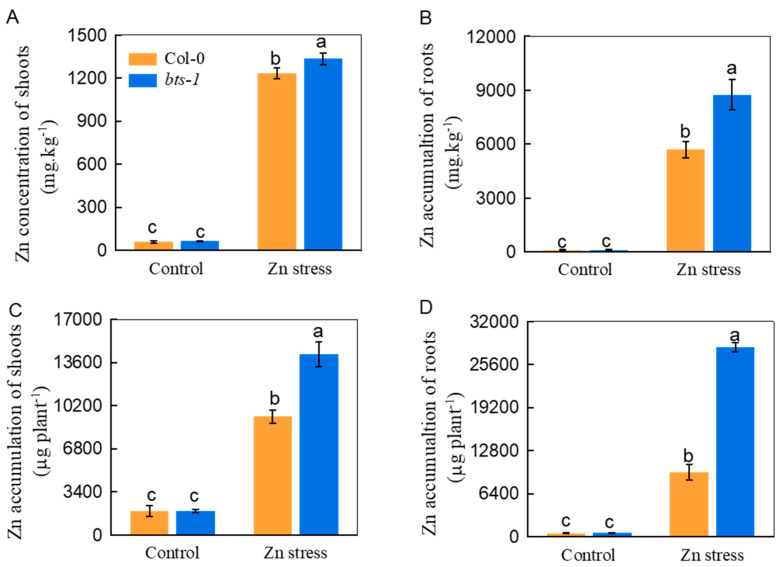
Zn concentration and accumulation in roots and shoots of wild-type Col-0 and *bts-1* mutants. The plants were treated as shown in Figure 2. (**A**,**B**) Zn concentration in shoots and roots, respectively. (**C**,**D**) Total Zn accumulation in roots and shoots were calculated according to the biomass per plant. Bars represent the standard deviation (*n* = 5). Different letters above the bars indicate significant differences (*p* < 0.05).

The biomass measurements were 88% and 55% for root and shoot increments of *bts-1,* respectively, compared with WT in the Zn-contaminated medium (Figure 2A,B). Additionally, the root elongation and chlorophyll concentration of *bts-1* were both clearly greater than those of WT plants in response to Zn stress (Figure 2C,D). Generally, improved Zn tolerance is accompanied by reduced Zn uptake by plants. Unexpectedly, the *bts-1* mutant had 53% higher and comparable Zn concentrations in roots and shoots, respectively (Figure 3A,B), compared with those of the wild-type plants. Moreover, both the shoots and roots of *bts-1* exhibited increased Zn accumulation, approximately 53% and 193% higher than that in the WT, respectively (Figure 3C,D). The above results suggest that *BTS* knockdown may provide a possible candidate method for improving the phytoremediation efficiency of Zn-contaminated soil.

### 3.2. Effect of BTS Inhibition on the Whole-Genome Transcriptome Profile under Zn Stress

**Figure 4:**
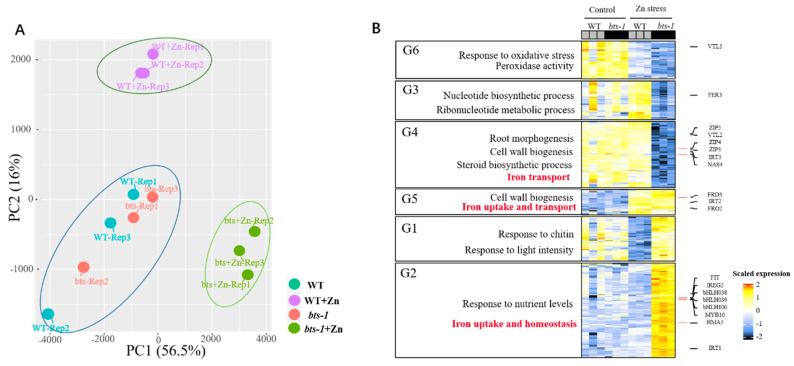
Global transcriptome analysis of wild-type Col-0 and bts-1 mutants. The treatments were similar to Figure 1. (**A**) Principal Component Analysis (PCA) of the transcriptional characteristics of WT and bts-1. (**B**) Heat map clustering and GO analysis of differentially expressed genes.

**Figure 5:**
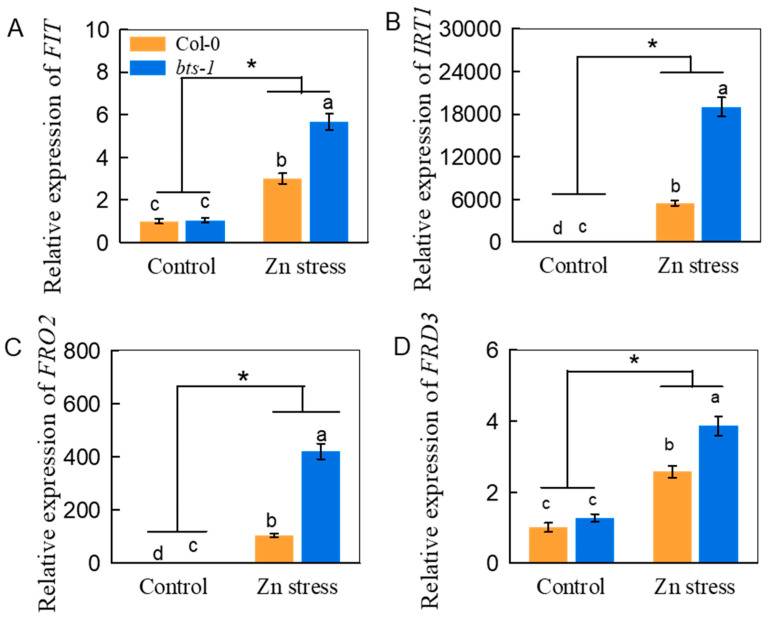
Effect of Zn stress on the expression of Fe nutrition-related genes in roots of wild-type Col-0 and *bts-1* mutants. Treatments were similar to Figure 1. (**A**–**D**) Expression of *FIT*, *IRT1*, *FRO2,* and *FRD3* in roots. Bars represent the standard deviation (*n* = 5). Different letters above the bars indicate significant differences between genotypes (*p* < 0.05). An asterisk indicates the significant interaction of gene expression between Zn treatment and genotype (*p* < 0.05).

To study the mechanism of improved Zn tolerance by *BTS* inhibition, we conducted RNA-sequencing (RNA-seq) analysis in the roots of Col-0 and *bts-1*. PCA analysis based on RNA-seq showed that the whole-genome transcriptome profile of *bts-1* mutants was similar to that of WT under control conditions. However, Zn stress treatment resulted in a significant difference in the whole-genome transcriptome profiles between WT and *bts-1* (Figure 4A). We then screened for the differentially expressed genes (DEGs) [−1 > log (fold-change) > 1; false discovery rate (FDR) < 0.05]. All DEGs were divided into six groups using hierarchical clustering (Figure 4B). Gene ontology (GO) analysis showed that several DEGs in the G2, G4, and G5 groups were related to ion homeostasis, particularly Fe homeostasis (Figure 4B). In detail, the Fe nutrition-related key genes, including *FIT*, *bHLH038*, *bHLH039*, *bHLH100*, *IRT1*, *FRO2, FRD3,* and *MYB10* were significantly upregulated in the *bts-1* mutants compared with WT under Zn stress. Among these genes, *FIT*, *IRT1*, *FRO2*, and *FRD3* were also re-examined by qRT-PCR, because these genes have been demonstrated to be the key genes involved in Fe uptake and transport. Similarly, Zn stress induced higher expression of these four genes in *bts-1* than in the WT (Figure 5A–D), thus confirming the RNA-seq data. The above results imply that the enhanced Zn tolerance in *bts-1* may be associated with the upregulation of genes responsible for Fe uptake and transport.

### 3.3. The Role of Fe Nutrition in BTS Inhibition-Improved Zn Tolerance

**Figure 6:**
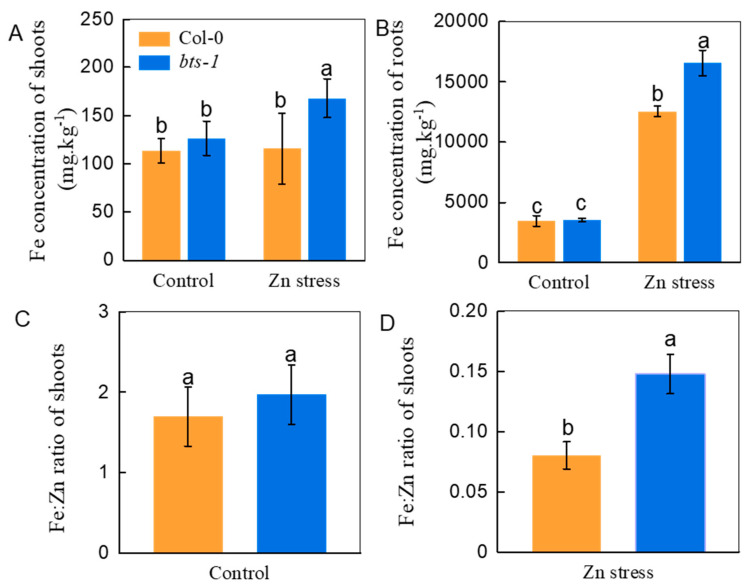
Fe concentration and Fe/Zn ratio in wild-type Col-0 and *bts-1* mutants. Treatments were similar to those shown in Figure 3. (**A**,**B**) Fe concentration in shoots and roots, respectively. (**C**,**D**) Fe: Zn ratio in shoots under normal and 300 µM ZnCl2 conditions, respectively. Fe:Zn ratio was calculated using Fe concentration and Zn concentration. Bars represent the standard deviation (*n* = 5). Different letters above the bars indicate significant differences (*p* < 0.05).

**Figure 7:**
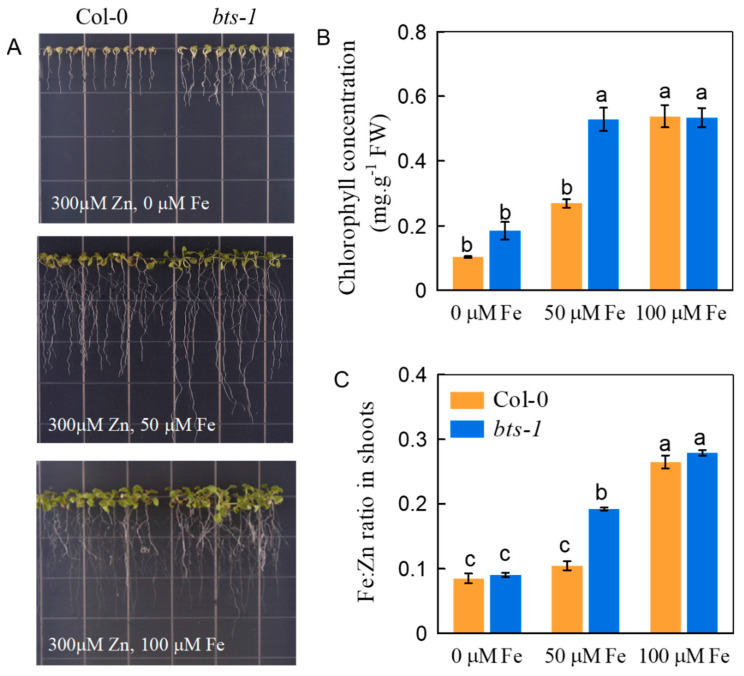
Effect of Fe on Zn tolerance of wild-type Col-0 and *bts-1* mutants. The three-day-old seedlings were transferred to agar medium and treated with 300 µM ZnCl_2_ and with different doses of Fe-EDTA (0, 50, and 100 µM). The assays were performed after 8 days. (**A**) The growth phenotype of plants. (**B**) The chlorophyll concentration. (**C**) The Fe/Zn ratio. Bars represent the standard deviation (*n* = 5). Different letters above the bars indicate significant differences (*p* < 0.05).

To clarify whether the increased Zn tolerance and accumulation in the *bts-1* mutant are associated with improved Fe nutrition in plants, the Fe concentration in plants was determined. Both the roots and shoots of *bts-1* showed a higher Fe concentration than that of wild-type plants when excess Zn was supplied into the growth medium, although there was no significant difference under control conditions (Figure 6A,B). As mentioned above, excessive Zn could replace Fe, which is involved in the proper functioning of a number of photosynthetic enzymes, thus playing a role in Zn toxicity. This indicates that the Fe/Zn ratio may be a key factor affecting the Zn tolerance of plants. Therefore, we compared the Fe/Zn ratios of the WT and *bts-1*. As expected, *bts-1* had a higher Fe/Zn ratio of in shoots compared with the WT under Zn toxicity conditions, while there was no obvious difference in normal growth medium (Figure 6C,D). Next, the growth of WT and *bts-1* was compared under Zn stress with a series of Fe doses. Both the WT and *bts-1* suffered severe Zn toxicity when the Fe was removed from the growth medium (Figure 7A), and as a result, the difference in either the growth or the chlorophyll concentration between the two plants was minimized (Figure 7A,B). With the increase of Fe in the growth medium, either growth or chlorophyll concentration in both WT and *bts-1* gradually increased. When the level of Fe was supplied at or over 100 µM, either the growth or the chlorophyll concentration of both plants was increased to a similar level (Figure 7A,B).

We then assayed the Fe/Zn ratio in three different Fe dose treatments, including 0 µM, 50 µM, and 100 µM. The results showed that the Fe/Zn ratio increased in both the WT and *bts-1* in response to an increased Fe supply (Figure 7C). In addition, the shoots of *bts-1* had similar Fe/Zn ratios to those of the WT treated with either 0 µM or 100 µM Fe, but had higher Fe/Zn ratios than those of the WT treated with 50 µM Fe treatment under Zn-contaminated conditions (Figure 7C). Notably, these Fe/Zn ratios across the various genotypes and Fe dose treatments were positively correlated with either growth or the chlorophyll concentration in the Zn-stressed conditions, and a higher Fe/Zn ratio could alleviate the toxicity of Zn to plants.

### 3.4. Knockout of BTS-Regulated FRO2 or FRD3 Leads to Impaired Zn Tolerance

**Figure 8:**
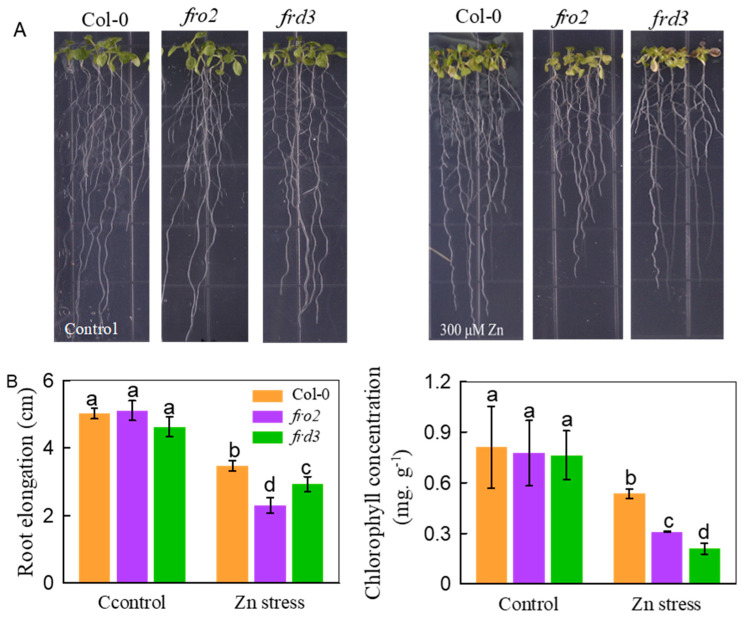
The tolerance of *fro2* and *frd3* mutants to Zn stress. The three-day-old seedlings of wild-type Col-0, *fro2*, and *frd3* mutants were transferred to agar medium and treated with 300 µM ZnCl_2_. The assays were performed after 8 days. (**A**) The growth phenotype. (**B**) The root elongation. The chlorophyll concentration. Bars represent the standard deviation (*n* = 5). Different letters above the bars indicate significant differences (*p* < 0.05).

We then further investigated the role of upregulated genes related to Fe uptake and transport in mediating the *BTS* knockdown-conferred Zn tolerance by using related mutants. *FRO2* is the dominant gene responsible for the reduction of ferric chelates into ferrous iron for subsequent uptake by *IRT1* [42]. Therefore, the *fro2* mutant was used to study the effect of Fe uptake genes on Zn tolerance. As shown in Figure 8, the *fro2* mutants exhibited more severe root growth inhibition and leaf chlorosis than the WT under Zn stress conditions. As a citrate effluxer, *FRD3* transports Fe to aerial parts by facilitating citrate efflux [42]. Therefore, the *frd3* mutant was used to further explore whether Fe transport from the roots to the shoots participates in the regulation of Zn tolerance. Similarly, *frd3* mutants suffered more serious toxicity than WT plants under Zn stress conditions (Figure 8). These results support our hypothesis that genes related to Fe uptake and transport play an important role in mediating *BTS* knockdown-conferred Zn tolerance.

## 4. Discussion

Because of their abilities in uptake and the root-to-shoot transport of heavy metals, plants can be exploited to help remediate polluted soil, which consequently reduces the harmful effects of heavy metals on food safety and human health [43,44]. In the past decades, phytoremediation has attracted great attention owing to its non-invasive, cost-effective, convenient, and environmentally friendly features. The efficiency of phytoremediation is highly dependent on both the uptake and tolerance of plants to heavy metals, which is reflected in the concentration of heavy metals and biomass production of plant organs, particularly in the shoot organs. Therefore, identifying biological mechanisms that can be used to increase Zn accumulation by enhancing Zn tolerance is necessary. The present study demonstrates that the inhibition of *BTS* expression using biotechnological pathways may provide a new strategy for enhancing the efficiency of Zn phytoremediation.

As a fine-tuning regulator of metal ions, partial disruption of the function of BTS was demonstrated to enhance the Fe nutrition in plants [28,29]. In addition to Fe, *BTS* mutants also have increased concentrations of other metal elements, e.g., Cd and Mn [45,46], mainly because these metal ions generally share the same transporters that are regulated by BTS. Accordingly, the roles of BTS in metal micronutrient biofortification or heavy metal tolerance deserve further exploration. In this study, through the RNA-seq analyses of transcriptome data, we showed that the Fe nutrition-related genes (*FIT, bHLH038, bHLH039, bHLH100, IRT1, FRO2, FRD3,* and *MYB10*) were also significantly upregulated in *BTS* knockdown mutants compared to the WT exposed to Zn stress. Interestingly, the upregulation of Fe nutrition-related genes due to the disruption of BTS was much higher under Zn stress conditions than under normal Zn supply conditions. Therefore, stress could intensify the upregulation of Fe nutrition-related genes by knocking down the *BTS* gene. Elements that possess similar physical and chemical properties can biologically antagonize each other [47,48], and excessive Zn can disrupt the key physiological processes through competing Fe-binding sites and causing growth defects in plants [16,27,47]. Therefore, the increased expression of genes related to Fe nutrition in the *bts-1* mutants rather than in Col-0 seedlings is most likely due to higher Zn accumulation in the former plants. This idea is supported by the observation that the root Zn concentration in the *bts-1* mutants was much higher than that in Col-0 seedlings.

Consistent with the expression profile of Fe nutrition-related genes, Zn stress improved Fe nutrition in roots, and the improvement was more obvious in the *bts-1* mutants than in the -0 seedlings. Because of the competition between Fe and Zn (i.e., the antagonism between Fe and Zn), the *BTS-*knockdown-induced improvement of Fe nutrition could be therefore expected to enable plant defenses against the toxicity of excess Zn. This view was supported by the observation that, although the *bts-1* mutants had a higher Zn accumulation level, their Zn tolerance was better than that of the wild-type plants (Figure 2A,B). The above results also suggested that the enhanced Zn tolerance and accumulation in the *bts-1* mutants was largely dependent on increased Fe concentration. This concept was further verified by the finding that the differences in Zn tolerance between WT and *bts-1* plants were minimized by either removing Fe or supplying excessive Fe in the growth medium (Figure 7A). In addition, the finding that knocking out the *BTS*-regulated genes related to Fe uptake and transport resulted in impaired Zn tolerance provides further support for the above notion.

As mentioned above, excessive Zn supply could lead to Fe deficiency and further inhibit plant growth. Therefore, iron homeostasis must be strictly controlled to avoid potential toxicity caused by the displacement of Zn in plants. Specifically, a proper Fe/Zn ratio is necessary for plant growth under conditions of Zn toxicity. The present study also indicates that *BTS* knockdown mutants displayed a higher Fe/Zn ratio in shoots compared with WT, which might be helpful for maintaining a healthy and balanced metal ion homeostasis. Accordingly, this would accordingly enhance the Zn tolerance of plants. Consequently, the tunable control of Fe uptake and transport regulation networks will be beneficial for improving Zn tolerance in plants. Here, we proposed that partial-loss-of BTS function could provide an attractive strategy for developing plant lines with enhanced efficiency for Zn phytoremediation.

## 5. Conclusions

The present study revealed that, upon Zn stress, partial loss of BTS function could elevate Fe level in *Arabidopsis* plants by upregulating the expression of several Fe uptake and transport-related genes, thereby increasing the Fe/Zn ratio in plant tissues. As a result, the tolerance to Zn stress was improved in the *BTS* knockdown mutants, while the WT plants experienced more serious Zn toxicity as a result of unbalanced metal ion homeostasis. Generally, Zn-contaminated soil also contains multiple heavy metal pollutants in the natural environment; therefore, it is meaningful to identify key genes that maintain the homeostasis of multiple metal ions in plants. This study provides novel insights into the improvement of phytoremediation efficiency by using genetic modifications.

## Figures and Tables

**Table 1:** Primers used in this work.

Usage	Gene Name	Forward Primer Sequence 5′-3′	Reverse Primer Sequence 5′-3′
Real Time PCR	*FIT*	CAGTCACAAGCGAAGAAACTCA	CTTGTAAAGAGATGGAGCAACACC
	*IRT1*	GAATGTGGAAGCGAGTCAGCGA	GATCCCGGAGGCGAAACACTTA
	*FRO2*	GATCGAAAAAAGCAATAACGGTGGTT	GATGTGGCAACCACTTGGTTCGATA
	*FRD3*	TGGACGATCATCCTCTTCATC	GGCAGAGGGCTCCATATTTT
	*BTS*	ATGCGAGCATTACAAGCGTAAC	GCATACAGAGCATTTCCGTCAC
Cloning	*BTS-promoter*	GCTATGACCATGATTACGAATTCATACGGCATG GAACGTTTCT	AAATCTGGTAACGGCGTCGCCATTTCCCCCAAAGCTTATCT
	*BTS*	AGATAAGCTTTGGGGGAAATGGCGACGC CGTTACCAGATTT	TCGCCCTTGCTCACCATGTCGACGGATGAGGTTGAGCAGTCCG
Genotyping	*BTS*	CCAAATGCGTTCGTAGGTAAG	TCAGATTTACACAAATTTGCAGC
	*LB1.3*	ATTTTGCCGATTTCGGAAC	

## Supplementary Materials

The following are available online at https://www.mdpi.com/article/10.3390/life12020216/s1, Figure S1: Identification of BTS knockdown mutant *bts-1*; Figure S2: Phenotypes of plants under series of Zn concentration conditions. After germinating on basal agar medium and growing for 3 days, transferring the seedlings to normal or Zn-contained medium for growing another 5 days, observing the growth of plants.
