# Supplementary material for: Inhibition of BRUTUS Enhances Plant Tolerance to Zn Toxicity by Upregulating Pathways Related to Iron Nutrition
Source: Life (Basel). 2022 Jan 30;12(2):216. doi: 10.3390/life12020216 (PMC8879508; doi:10.3390/life12020216)
Supplement: Supplementary file 1 [file life-12-00216-s001.zip › life-1537130-supplementary.pdf]

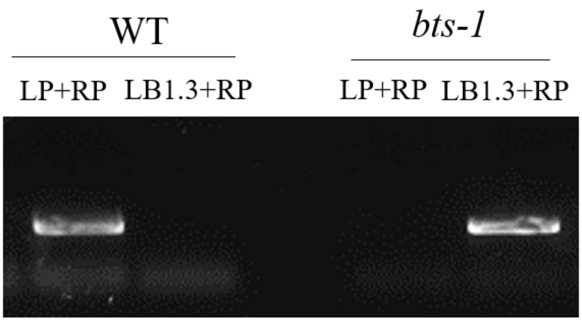

Figure S1. Identification of BTS knockdown mutant *bts-1*.

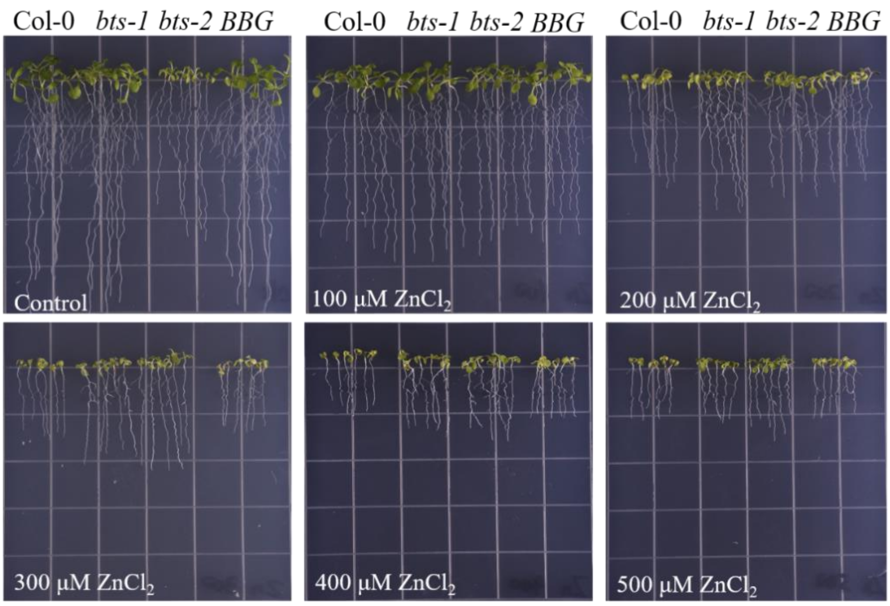

Figure S2. Phenotypes of plants under series of Zn concentration conditions. After germinating on basal agar medium and growing for 3 days, transferring the seedlings to normal or Zn-contained medium for growing another 5 days, observing the growth of plants.
